# Evaluation of respiratory syncytial virus (RSV) vaccine eligibility in an outpatient adult infectious diseases clinic

**DOI:** 10.1017/ash.2026.10342

**Published:** 2026-05-19

**Authors:** Amy Liu, Conor Stack, Monica V. Mahoney

**Affiliations:** 1 Boston Medical Center, USA; 2 Beth Israel Deaconess Medical Center, USA

## Abstract

Respiratory syncytial virus (RSV) vaccination access is complicated by insurance coverage limitations. This two-week study showed that at least 75% of eligible older adults were covered by Medicare Part D, which restricts vaccine administration to retail pharmacies. This increases complexity of care coordination, may decrease vaccination rates, and highlights the need for expanded vaccine coverage.

## Background

Respiratory syncytial virus (RSV) is a respiratory virus impacting older adults and infants. Older adults are at increased risk of severe disease and hospitalizations due to concomitant conditions and weakened immune response.^1^ Three RSV vaccines are approved for use in the adult population. The Advisory Committee on Immunization Practices (ACIP) recommends RSV vaccination in all adults aged ≥ 75 and individuals aged 50–74 with conditions increasing the risk of severe RSV disease.^2,3^ The use of RSV vaccines in pregnant patients is beyond the scope of this study. During the 2023–2024 RSV season, real-world data demonstrated RSV vaccination was ≥70% effective in preventing RSV-associated hospitalizations and emergency department visits in patients aged ≥ 60.^4^

Commercial insurance plans, Medicaid, and Medicare are required to cover ACIP recommended vaccines.^5^ However, how plans cover vaccines varies. Vaccines covered under medical benefits can be administered in ambulatory clinics. Vaccines covered under pharmacy benefits should be administered at retail pharmacies; otherwise, patients may be responsible for the cost. For Medicare patients, the RSV vaccine is solely covered through Part D (pharmacy) benefits. In contrast, Medicare covers other respiratory viral vaccines (influenza and COVID-19) under Part B (medical) benefits. Commercial and Medicaid insurances typically cover RSV vaccines under both medical and pharmacy benefits. At study time, the RSV vaccine was only recommended for patients aged ≥ 60. We aimed to assess the percentage of patients eligible for the RSV vaccine who required an additional healthcare visit (retail pharmacy) for receipt.

## Methods

This single-center retrospective study was conducted from 2 September 2024 to 13 September 2024. All patients with any appointment at the adult Infectious Diseases outpatient clinic at Beth Israel Deaconess Medical Center (Boston, MA) during the study period were screened for inclusion. Patients aged ≥ 60 years were included. A manual chart review of the electronic health record was conducted to identify age, race/ethnicity, insurance coverage, RSV vaccination status, and comorbid conditions. Comorbid conditions were collected for patients aged 60–74 based on ACIP recommendations: history of cardiovascular disease, end stage renal disease, diabetes mellitus with end-organ damage, lung disease, severe obesity, neurologic conditions, hematologic disorders, moderate to severe immunocompromise, and residence in a long-term care facility.^2^ Baseline comorbid conditions were not collected for patients aged ≥ 75 as they were eligible for vaccination based on age. Vaccination status was determined via the state immunization information system and EHR records. Previous vaccination, age, and comorbid conditions were the only vaccine eligibility criteria collected.

The primary outcome of this study was percentage of eligible patients aged 60–74 and patients aged ≥ 75 who required vaccination at a retail pharmacy. Descriptive statistics were performed for baseline characteristics and outcomes.

This study was approved by the institutional review board with exempt status (protocol #2024P000693). A second investigator reviewed 5% of patients to ensure data accuracy.

## Results

There were 242 patients with appointments in the Infectious Diseases clinic during the study period. Thirty-seven patients were aged ≥ 75, and 92 patients were aged 60–74. Patient demographics are presented in Table 1. Medicare was the most prevalent insurance.

**Table 1.** Patient Demographics

Baseline characteristics of patients aged 60+ Years (n = 129)	
Sex, n (%)	
Male	68 (53)
Female	61 (47)
Age (years), Median (IQR)	70 (65–76)
**Age ≥75, n (%)**	37 (28.7)
**Age 60-74, n (%)**	92 (71.3)
Race, n (%)	
White	83 (64)
Black	19 (15)
Asian	11 (9)
Other	16 (12)
Ethnicity, n (%)	
Non-Hispanic	124 (96)
Hispanic	5 (4)
Insurance, n (%)	
Medicare	98 (76)
Commercial/private	21 (16)
Medicaid	9 (7)
None	1 (1)
Comorbid conditions of patients ages 60–74, n (%) n = 92*	
Cardiovascular disease	21 (23)
Lung disorders	33 (36)
Diabetes with end organ damage and/or on an SGLT2i/insulin	17 (18)
Immunocompromised	12 (13)
Liver disorders	7 (8)
ESRD/HD	5 (5)
Neurologic disorders	3 (3)

ESRD, end-stage renal disease; HD, hemodialysis; IQR, interquartile range; SGLT2i, sodium-glucose cotransporter-2 inhibitors.

*Percents add up to greater than 100% as patients may have more than 1 co-morbidity.

Among patients aged ≥ 75, 24/37 (64.9%) never received the RSV vaccine, and 20/24 (83.3%) were enrolled in Medicare. Among ages 60–74, 60/92 (65.2%) never received the RSV vaccine, with 38/60 (63.3%) having ≥1 comorbid condition conferring eligibility. Further, 29/38 (76.3%) were enrolled in Medicare.

## Discussion

To our knowledge, this is the first study assessing real-world RSV insurance coverage on vaccine utilization and eligibility. A convenience snapshot of 2 weeks during respiratory viral season in September was chosen. Approximately two-thirds of eligible patients in both cohorts had yet to receive the RSV vaccine, and most eligible individuals were enrolled in Medicare. These patients would be unable to receive the RSV vaccine in clinic given coverage under Part D (pharmacy) benefits rather than Part B (medical) benefits. Patients would have to go to retail pharmacies or pay out-of-pocket—approximately $350.^6^ This creates both direct and indirect healthcare costs, increases barriers to care, and disproportionately affects older patients. While pharmacists are the leading immunizers in the United States, clinic vaccinations are more often due to clinician recommendation (19.1% pharmacies vs 61.3% clinics)—an important distinction with newer vaccine products.^7^ Immunization practice standards include recommending and offering vaccines at the same visit, increasing vaccine acceptance rates.^8^

This was a single-center, specialty clinic, retrospective study with a limited duration, which may not fully capture RSV vaccine utilization or reflect other clinic practices. Clinic appointment indications were not assessed; therefore, some patients may have been ineligible for vaccination due to acute illness. Lastly, there was only a 1-year period from first RSV vaccine approval to study conduction, limiting time for vaccination.^9^

The ACIP recommendations originally included “shared clinical decision-making” in adults aged ≥ 60.^2^ In August 2024, recommendations were simplified, citing all adults ≥75 and adults 60–74 with certain comorbid conditions receive RSV vaccination.^2^ In July 2025, RSV vaccine eligibility was further decreased to adults ≥50 with certain comorbid conditions.^3^ This expands the pool of eligible patients, introduces more commercial insurance plans, and may increase confusion among patients and clinicians, leading to forgoing vaccine recommendations and decreasing vaccine acceptance rates. While clinics may apply to be an “out of network” provider and bill under Medicare Part D, the process is tedious and convoluted. A simpler solution would be for all recommended vaccines to be covered at all healthcare locations, eliminating the bifurcation of medical versus pharmacy benefit coverage.
